# A newly designed anatomical plate for the therapy of posterolateral tibial plateau fracture via a supra-fibular-head approach: a retrospective study

**DOI:** 10.1038/s41598-024-62227-4

**Published:** 2024-05-22

**Authors:** Xiaoji Zhou, Jiangshan Zhou, Huajun Qian, Deping Zhan, Chunxiao Qian, Lv Pan, Xudong Chu

**Affiliations:** Department of Orthopedics, Affiliated Huishan Hospital of Xinglin College, Nantong University, Wuxi Huishan District People’s Hospital, Wuxi, Jiangsu China

**Keywords:** Musculoskeletal system, Trauma, Orthopaedics

## Abstract

The posterolateral tibial plateau fracture is a special type of intra-articular fracture, for which there is no simple, safe, and effective standardized procedure. In this paper, we evaluate the clinical efficacy and the advantages of the treatment of posterolateral tibial plateau fracture by using our designed proximal lateral tibial rim plate for the posterolateral condyle of the tibial plateau via the space above the fibula head. Thirty-eight patients with posterolateral tibial plateau fractures from June 2018 to June 2021 were retrospectively analyzed. CT scans were used to classify the degree of injury in the included patients. All of them were fixed with reduction using an approach above the fibula head combined with a homemade anatomical plate. The regular postoperative review was performed to instruct functional knee exercises. Postoperative complications were observed and follow-up visits were performed to assess the functional outcome. A total of 38 patients with posterolateral tibial plateau fractures, 13 males and 25 females were included in the study. All patients were followed up for 13–26 months, with a mean of 15.3 months. There were no postoperative complications such as numbness of the limb, knee joint instability, etc. X-ray review showed that the fractures were all healed, and the healing time was 10–16 weeks, with an average of 12.1 weeks; none of the internal fixation loosening and loss of articular surface occurred during the follow-up period. At the last follow-up, according to the HSS knee function score criteria, the scores were 79–98, with an average of 91.3. The HSS score presented excellent in 34 cases (89%) and good in 4 cases (11%). The Rasmussen score was graded as excellent in 29 cases (76%) and good in 9 cases (24%). In conclusion, The treatment of posterolateral tibial plateau fractures by an approach above the fibula head has the advantages of simplicity and safety, small trauma, and no risk of vascular and nerve injuries, and the anatomical proximal lateral tibial rim plate can play a direct and effective supporting role for the bone fragments of the posterolateral condyle, and the combination of both of them has obvious advantages in the treatment of posterolateral condylar fracture of the tibial plateau, and it is a method worth borrowing and popularizing.

## Introduction

Tibial plateau fractures have tended to be more prevalent due to the escalation of traffic accidents, which are mostly caused by a combination of axial forces and varus or valgus to the knee^1,2^. Tibial plateau fractures involving the posterolateral quadrant account for 7–15% of tibial plateau fractures approximately and are among the most difficult to treat^3^, as the posterolateral fragments are usually obstructed by the fibular head and posterolateral corner structure^4^, and various neurovascular bundles (the popliteal artery/vein, the tibial nerve, the common peroneal nerve, etc.) run across the posterolateral region. These have led to various surgical approaches aimed at exposing and fixing this fracture. Currently, the mainstream options that have been described to reduce and fix the posterolateral fragments are trans fibular neck osteotomy approach, posterolateral approach, and posterior approach, which could allow direct visualization of the posterolateral fragment and the application of an antiglide or buttress plate for fixation. However, these approaches that involve the dissection of intricate structures have a high risk of iatrogenic injury to the neurovascular bundle, thereby increasing the operative difficulty as well^5–7^.

**Figure 1:**
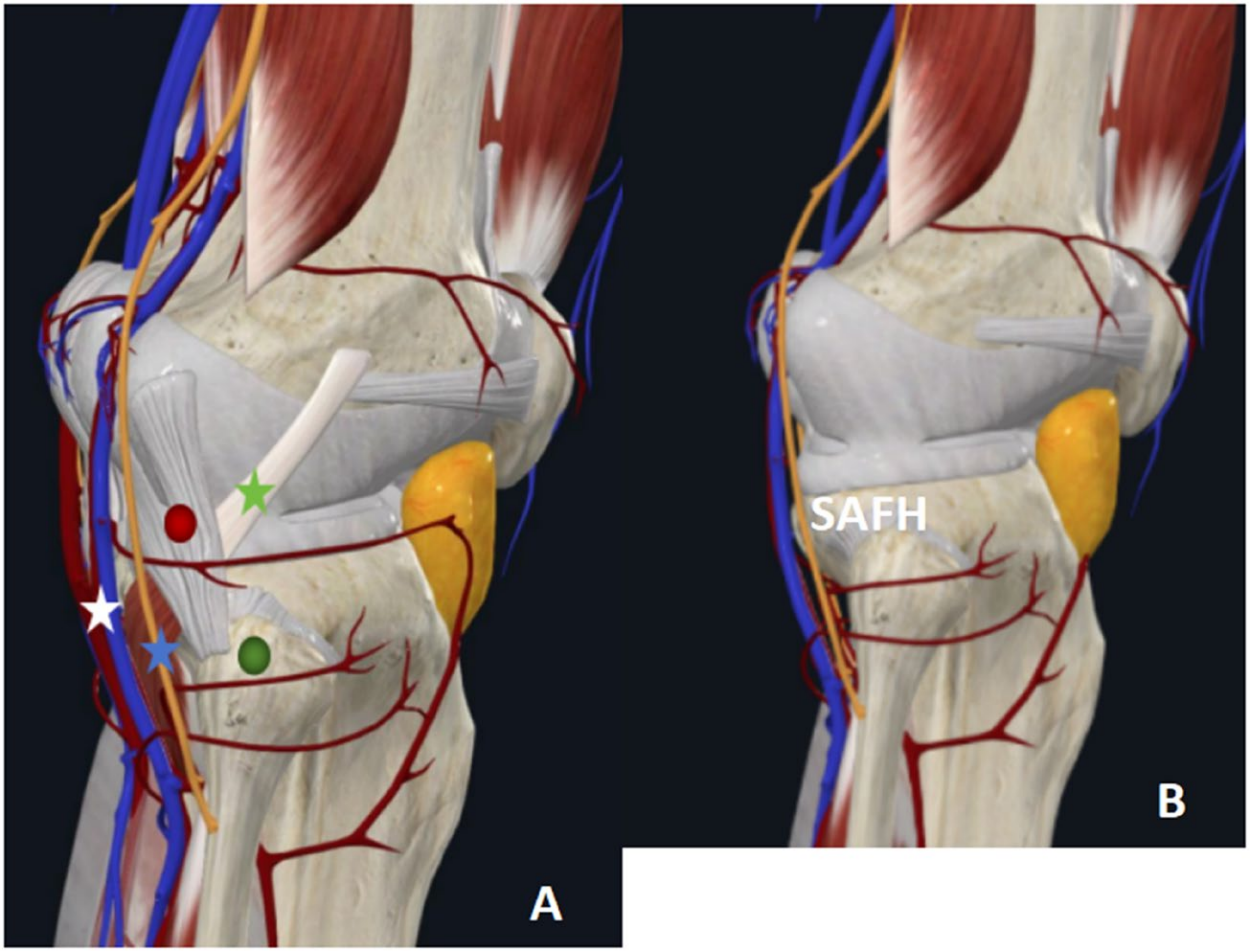
A three-dimensional illustration showing the lateral tibial plateau. Lateral views showing the anatomical relationship (**A**). The SAFH can be observed after retracting the fibular collateral ligament and hamstring tendon posteriorly (**B**). Green dot, caput fibulae; Red dot, fibular collateral ligament; Blue star, common peroneal nerve; Green star, musculus popliteus; White star, vena poplitea, and arteria poplitea; SAFH, space above the fibula head.

**Figure 2:**
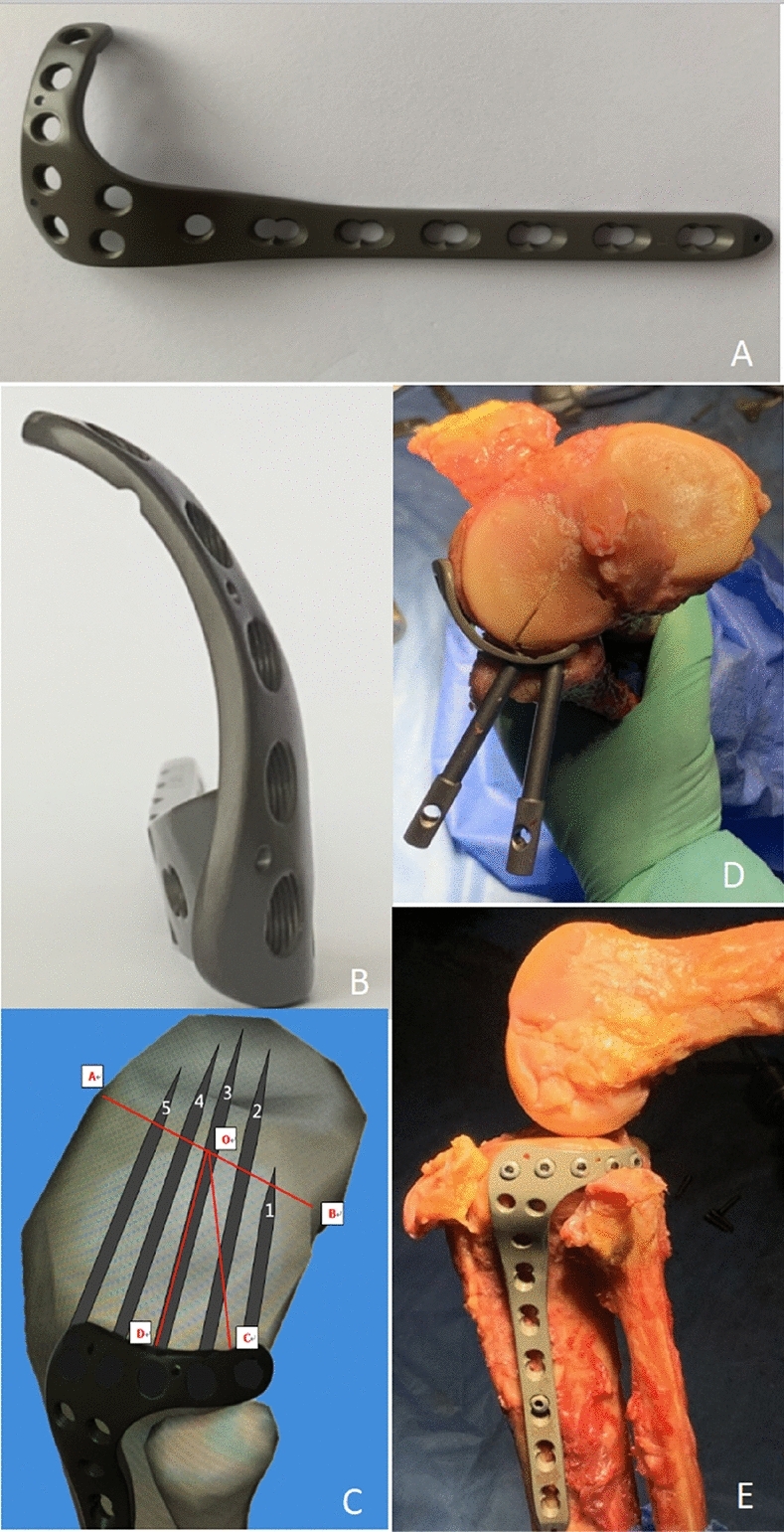
(**A**) Our anatomical proximal lateral tibial rim plate with an L-shape with five holes on the horizontal arm. (**B**) A view of the head of the anatomic plate. The head of the anatomic plate is a curved structure that can encompass the entire lateral tibial plateau. (**C**) A schematic diagram showing the raft fixation by this anatomic plate above the fibular head. Mark A is the center of the leading edge of the tibial plateau, mark B is the center of the trailing edge of the tibial plateau, mark O is the center of the tibial intercondylar eminence, mark C is the posterior margin of the articular surface of the fibular head, and mark D is the anterior edge of the articular surface of the fibula head. BOC represents Area I, DOC represents Area II, and AOD represents Area III. Screw 1 and 2 can provide effective subchondral support for the posterolateral bone fragments (Area I), and the other three screws can properly fix the fragments in Area II and Area III simultaneously. (**D**,**E**) Fresh specimen autopsy showing the simulated implantation of this anatomic plate and screw.

Therefore, through the anatomical study of the knee joint and the measurement of imaging-related data, we proposed the space above the fibula head for the first time in China in 2012 and designed and produced an anatomical plate that was compatible with the space above the fibula head (Figs. 1, 2). From June 2018 to June 2021, 38 cases of posterolateral condylar fractures of the tibial plateau were treated by using the supra-fibular-head approach combined with a homemade anatomic plate, and satisfactory clinical results were achieved. This retrospective study aims to introduce the surgical method of the supra-fibular-head approach combined with a homemade anatomical plate for the treatment of posterolateral condylar fracture of the tibial plateau; to assess the clinical efficacy of this surgical approach; and to explore the advantages of this surgical approach in the treatment of posterolateral condylar fracture of the tibial plateau.

## Materials and methods

### Case inclusion

This retrospective study included a total of 38 patients (13 males and 25 females) diagnosed with posterolateral quadrant fractures of the tibial plateau, aged 30–73 years (average 51.47 ± 9.67 years), treated in our Department of Orthopedics from June 2018 to June 2021. All patients underwent open reduction with internal fixation via a trans-fibular-head approach using our designed anatomical locking plate at our institution.

The inclusion criteria include: (1) posterolateral quadrant fractures of the tibial plateau as revealed by imaging examinations; (2) posterolateral fragments depressed and displaced beyond 3 mm; (3) treatment with open reduction and internal fixation via the supra-fibular-head approach using our novel plate; (4) complete case data was available; (5) age equal to or greater than 18 years.

The exclusion criteria included: (1) open fractures of the tibial plateau and pathological fractures; (2) fractures combined with vascular and/or nerve injury; (3) beyond 3 weeks between the injury and the initial operation; (4) inability to give informed consent; (5) general poor physical state.

All surgical operations were performed by 2 senior orthopedists (C.Q. and X.C.) who specialized in traumatic surgery. Independently, two authors reviewed the radiographic images of the anteroposterior and lateral views and CT reconstruction images during the follow-up period. Out of 38 cases, 15 cases suffered traffic accidents, 5 cases were injured in fall from height, and 18 cases were injured in fall hurt. All cases in this study were classified according to Schatzker’s Classification, the Orthopaedic Trauma Association Classification, and the three-dimensional CT partition of the lateral tibial plateau. Patient characteristics are summarized in Table 1. The study protocol was approved by the Institutional Review Boards and the Ethics Committees of Wuxi Huishan District People’s Hospital (Ethics No. 0001366), and all participants provided written informed consent. It was performed in accordance with relevant guidelines and regulations. The research involving human research participants has been performed in accordance with the Declaration of Helsinki.

**Table 1:** Summary of the main demographic data.

Basic characteristics		
Total patients	N = 38	%
Average age (range)	51.47 ± 9.67	
Gender		
Male	13	34.21
Female	25	65.78
Knee		
Left	26	68.42
Right	12	31.58
Cause of injury		
Traffic accident	15	39.47
Fall from height	5	13.16
Fall hurt	18	47.37
CT partition		
I	7	18.42
II	6	15.79
I + II	14	36.84
II + III	2	5.26
I + II + III	9	23.68
Schatzker classification		
I	6	15.79
II	16	42.11
III	3	7.89
V	7	18.42
VI	6	15.79
AO type		
B1	6	15.79
B2	3	7.89
B3	16	42.11
C2	5	13.16
C3	8	21.05
Comorbidity		
Osteoporosis	1	2.63

Anteroposterior and lateral X-ray and CT scans (axial, 2D, and 3D reconstruction) were performed for routine preoperative examinations of the knee joint. Before surgery, patients were immobilized using casts or calcaneal traction. To avoid deep venous thrombosis in the lower limb, patients were prescribed low molecular heparin sodium. Soft tissue condition was the basis for the surgery schedule.

### Features of our novel anatomical plate

Our newly designed anatomical plate (patent number: ZL201220528187.9) is in an inverted L-shape, with five holes on the horizontal arm forming an arc that encompasses the entire lateral tibial plateau, including Area I, Area II, and Area III. Besides, the horizontal arm of the plate can be used to create rafting constructs with multiple locking screws to provide subchondral support for fragments through the space above the fibular head. The plate is made of a titanium alloy (thickness, 2 mm; horizontal arm width, 8 mm). Five 3.5-mm screw holes are made in the horizontal arm; the 3.5-mm plate body can be set to different lengths according to the patient’s needs. Figure 2 shows the newly designed plate.

### Surgery procedure

#### Preparation and exposure

**Figure 3:**
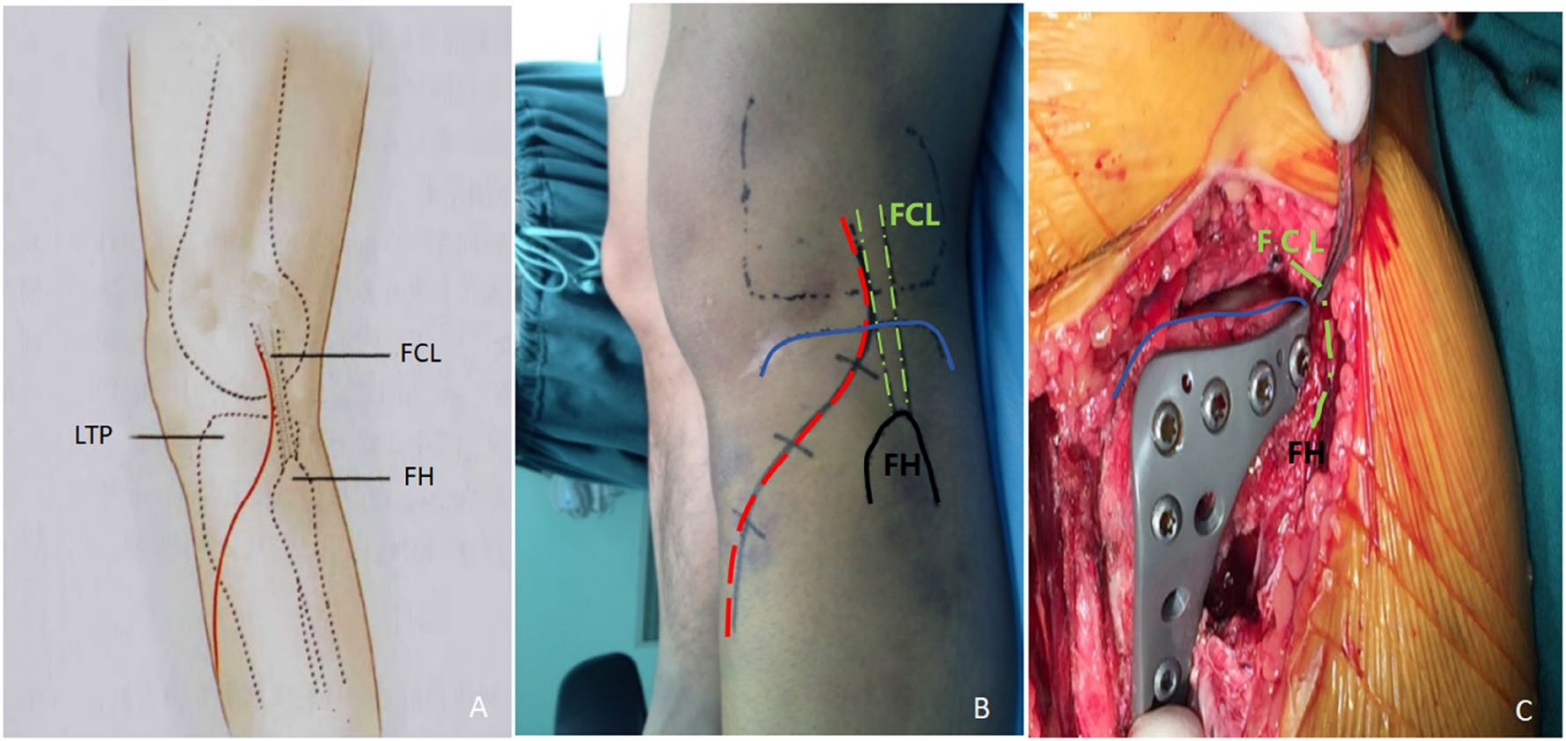
(**A**,**B**) An illustration of the incision and anatomic landmarks. Red line: skin incision. Blue line: the articular surface of the lateral tibial plateau. Green line: fibular collateral ligament. (**C**) Intraoperative image showed the implantation of our newly designed anatomic plate and screw. *FH* fibular head, *LTP* lateral tibial plateau, *FCL* fibular collateral ligament.

After anesthesia induction, the patients underwent surgery in a supine position with the injured limb maintained in a slightly flexed position. The superior segment of incision was performed closely along the anterior border of the fibular collateral ligament, starting 2 cm above the knee joint surface, extending along the anterior border of the fibular collateral ligament to the fibular head, and then extending anteriorly and distally to the Gerdy’s tubercle (Fig. 3). According to the needs of fracture fixation, it can extend downward along the lateral side of the tibia. The subcutaneous tissue and deep fascia were dissected to expose the anterior edge of the fibular collateral ligament, and then the termination of the tibialis anterior muscle was forward separated along the bone surface to expose the anterolateral plateau (Area III). The lateral plateau (Area II) could be exposed by the dissection of the space between the fibular collateral ligament and the lateral plateau rim with knee flexion to approximately 60°. Next, the fibular collateral ligament and hamstring tendon could be mobilized to retract posteriorly using a retractor, and the posterolateral condyle of the tibial plateau (Area I) could be fully exposed with supplementary manoeuvres of slight internal rotation and inversion of the knee joint. The inferior margin of the coronal ligament and joint capsule was incised horizontally above the lateral plateau, and a meniscus hook was used for traction of the meniscus. After clearing the hematoma in the articular cavity, most parts of the posterolateral articular surface were clearly visualized.

#### Restoration and fixation

A cortical window was created approximately 1 cm below the cartilage surface in front of the articular surface of the fibular head. With the help of posterolateral articular surface exposure through the space above the fibular head, the collapsed posterolateral articular fragments were carefully elevated from front to back through the cortical window with an osteotome until the articular surface was level. Multiple K-wires were used for temporary fixation. The cavity of bone defects was adequately filled with allogeneic bones. Then, our novel anatomical locking plate was used for fixation: the plate body adhered to the lateral surface of the proximal tibial, and the horizontal arm formed an arc that crosses the space above the fibular head and encompasses the entire posterolateral condyle of the plateau (Area I). Two posterior screws were inserted parallel to the articular surface to achieve the fixation of Area I, and the other 3 screws were for the simultaneous fixation of Area II and Area III.

### Post-operative protocol and assessment

A plaster cast was not used to immobilize the knee joint. To promote detumescence, a trapezoidal cushion was used to place the lower limbs. Antibiotics were routinely administered for 24 h after operation to prevent infection. To prevent venous thrombosis in the lower extremities, low molecular weight heparin sodium was given subcutaneously 12 h after surgery. Anteroposterior and lateral radiographs, as well as three-dimensional CT reconstruction of the knee joint, were performed 2 days after surgery to evaluate the articular surface reduction. On the first postoperative day, patients were instructed to undertake isometric contraction of the quadriceps femoris. Knee range of motion exercises were applied to allow less than 90° knee flexion within 6 weeks postoperatively and was increased gradually to 120° after 6 weeks postoperatively. Patients were encouraged to begin partial weight bearing with crutches at 8 weeks postoperatively. Full weight-bearing was authorized after the bony union had been radiographically confirmed to be stable enough.

The primary evaluations included the functional and radiographic outcomes. Hospital for Special Surgery (HSS), Short Musculoskeletal Function Assessment (SMFA) score and range of motion (ROM) were obtained to assess knee function and health at the final follow-up. HSS was evaluated according to the pain, walking, and standing function, range of motion, muscle strength, flexion deformity, and knee instability, all the patients were graded as excellent (≥ 85 points), good (70–84 points), fair (60–69 points) and poor (< 60 points)^8^.

Four radiographic parameters, namely, fracture healing time, radiological Rasmussen score, tibial plateau angle (TPA), and posterior slope angle (PSA), were evaluated by 2 operating clinicians based on the X-ray films and CT scans. The radiological Rasmussen score was evaluated according to the joint surface depression, condylar widening, and angulation (valgus/varus), each of which scored 6 points, with a total score of 18 points^9^. The tibial plateau fracture reduction was expressed as excellent (18 points), good (12–17 points), fair (6–11 points), and poor (< 6 points). In addition, a bony union was defined as a union of at least 3 cortices in anteroposterior and lateral views on follow-up radiographs^10^.

The secondary outcome parameters included the preoperative treatment time, operation time, blood loss, postoperative hospitalization duration, follow-up period, and postoperative complications.

### Statistical analyses

IBM SPSS statistics version 26.0 software (IBM Inc., Chicago, IL) was adopted for statistics. Baseline continuous variables were presented as the mean values standard deviation (SD) and range, and categorical variables were expressed as numbers and percentages (%). The Student–Newman–Keuls, multiple comparison tests, were used to examine the differences among the multiple groups. Differences between groups with unequal variances were examined by Dunnett’s T3 test. P < 0.05 was considered statistically significant.

## Results

### Clinical data

The average follow-up duration was 15.34 ± 4.36 months (range, 13–26 months). The average operation time was 105.5 ± 35.8 min (range 55–205 min), and the average intraoperative blood loss was 98 ± 115 ml (range 10–600 ml). The average treatment time before surgery was 5 ± 3 days (range 1–16 days), and the average hospitalization duration was 16 ± 5 days (range 9–30 days). All patients achieved fracture union after a mean of 12.05 ± 1.74 weeks (range 10–16 weeks) (Table 2).

**Table 2:** Summary of radiological and clinical outcomes.

	Average	Standard deviation	Range
Follow-up (months)	15.34	4.36	13–26
Time for fracture union (weeks)	12.05	1.74	10–16
The radiological Rasmussen	17.26	1.43	14–18
HSS score	91.32	5.45	79–98
SMFA score	21.16	6.97	10–36
Knee movement (°)			
Extension	0	0	0
Flexion	127.53	5.09	115–138

### Functional evaluation

Based on the assessment, at the final follow-up, the patients had a mean HSS knee function score of 91.32 (range 79–98) and a mean SMFA score of 21.16 (range 10–36). The HSS score presented excellent in 34 cases (89%) and good in 4 cases (11%). This was also shown by the average knee ROM 127.53° (range 115°–138°) based on the final assessment.

### Radiographic evaluation

The mean TPA was 90.97 ± 1.82°at 2 days post-operatively and 90.36 ± 2.33°at the last follow-up, all of which were significantly better than they were preoperatively (83.52 ± 4.10°) (P = 0.001). The PA was 7.90 ± 1.07°at 2 days post-operatively and 7.84 ± 1.41° at the last follow-up, all of which were significantly better than they were pre-operatively (19.85 ± 5.69°) (P = 0.001) (Table 3). Based on the assessment, the patients had a mean Rasmussen radiologic score of 17.26 (range 14–18). In addition, the Rasmussen score at the final follow-up was graded as excellent in 29 cases (76%) and good in 9 cases (24%).

**Table 3:** Variations in tibial plateau inversion and valgus angles.

Parameters	Pre-operation (range)	Post-operation (range)	Last-follow up (range)
TPA (°)	83.52 ± 4.10* (76.45–92.26)	90.97 ± 1.82 (86.58–95.60)	90.36 ± 2.33 (86.05–95.01)
PA (°)	19.85 ± 5.69* (12.88–26.84)	7.90 ± 1.07 (5.89–10.05)	7.84 ± 1.41 (5.22–10.45)

*Preoperative vs. last follow-up, P < 0.05; Preoperative vs. post-operation, P < 0.05.

**Figure 4:**
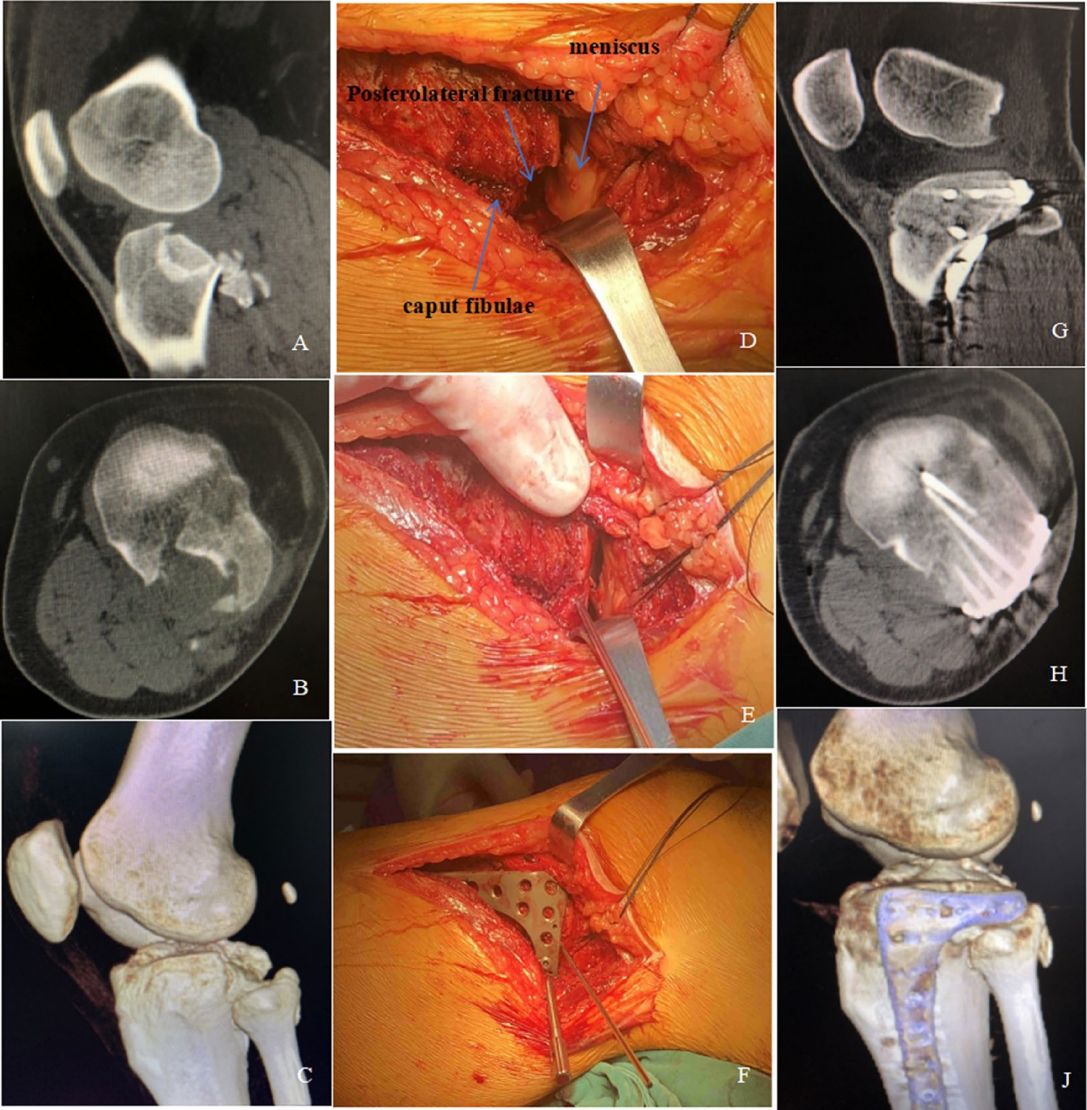
A case of type II tibial plateau fracture, male, 56 years old. (**A**–**C**) Preoperative CT showed a significantly depressed fragment in posterolateral plateau. The 3D reconstruction CT showed that the fracture involved the Area I and Area II of the lateral plateau. (**D**–**F**) Surgical exposure, reduction, and fixation of the posterolateral plateau. (**G**,**H**,**J**) Postoperative CT showed a satisfactory reduction of posterolateral condyle fracture, with proximal positioning of the subchondral screws (raft screws) to support posterolateral joint surface impaction injuries.

Typical case (Fig. 4): The patient is a 56-year-old male, who was admitted to the hospital with left knee pain and activity limitation for 1 h after a car accident. The 3D reconstruction CT showed that the left tibial plateau was fractured at the posterior lateral condyle, and the fracture involved the Area I and Area II of the lateral plateau. Under lumbar anesthesia, posterior lateral condylar fracture reduction, allograft bone grafting, and internal fixation with a homemade anatomical plate were performed above the head of the fibula, and the fracture was clinically healed 13 weeks after the operation, with a score of 18 points according to Rasmussen radiological criteria and 97 points according to the HSS knee function score.

### Complications

None of the patients had postoperative complications, including nerve injury, vascular injury, wound infection, fracture re-displacement, internal fixation failure, or bone nonunion. Lower extremity venous thrombosis occurred in three patient’s peri-operation. Two patients developed incision fat liquefaction and resolved with dressing changes after surgery.

## Discussion

Posterolateral fracture of the tibial plateau is characterized by high misdiagnosis, difficult exposure, difficult reduction, fixation, etc. Misdiagnosis and inappropriate choice of treatment can lead to serious joint deformity and functional impairment. Hence, management is quite technically demanding for an orthopedic surgeon because restoration of the rim continuity, anatomical reduction of the articular surface, and achievement of sufficient post-operative ligamentous stability and rigid internal fixation of fractured fragments are necessary to achieve functional rehabilitation^11,12^.

Recently, a three-dimensional CT image is necessary to establish morphological types of posterolateral fractures which is essential to decide on surgical approach and optimal plate. There are major morphological types of posterolateral tibial plateau fractures according to three-dimensional CT images: pure split, split depression, contained pure depression, and non-contained depression^13^. Currently, various surgical approaches have been reported, which can be grossly divided into two classes: anterior approaches and posterior approaches. The choice of approach mostly depends on the associated morphological types of fractures and the surgeon’s preference. Merits associated with the posterior approach and the posterolateral approach with or without fibular osteotomy include direct visualization and manipulation of posterolateral fracture fragments and adequate implant positioning, especially when buttressing is necessary^14^. However, the anterior tibial artery penetration point at the interosseous membrane was close to the posterolateral tibial plateau, the mean distance was 46 mm approximately, which may cause iatrogenic vessel rupture^15^. Moreover, the posterolateral approach with fibular osteotomy carries the risk of nonunion or malunion at the osteotomy site^7,16^. Furthermore, if cases were accompanied by split wedge fragments of the anterolateral tibial plateau, neither the posterior approach nor the posterolateral approach could be performed to manage anterolateral fragments. Other limitations also have been pointed out, including iatrogenic damage to the common peroneal nerve and the difficulty in plate removal surgery.

Although most orthopedic surgeons are familiar with the traditional anterolateral approach, the clinical application has been limited owing to the difficulty in providing full visualization or utilizing the buttress plate. To overcome these limitations, various forms of osteotomy were proposed to improve access to the posterolateral region of the tibial plateau. A submeniscal approach combined with lateral femoral epicondylar osteotomy described by Yoon et al.^17^ could increase intraoperative exposure, while a new approach (needs fibular head osteotomy) described by Yu was performed to expose and manipulate the posterolateral fragments^7^. We also have performed the feasibility study of plating posterolateral tibial plateau fractures via an anterolateral supra-fibular-head approach. According to our study, the space above the fibular capitellum allows direct visualization of the posterolateral and anterolateral articular surface and the possibility of placing the plate more posteriorly. Compared with the direct posterolateral and conventional anterolateral approaches (needs osteotomy), the anterolateral supra-fibular-head approach can provide a safer space around the rim of the posterolateral tibial plateau and reduction of the articular surface could be performed under direct visualization or through the anterolateral cortical window. Even more important advantages of this approach, perhaps, are its ease of exposure, and decreased likelihood of iatrogenic injury to the neurovascular structures. However, it is noteworthy that this approach is limited when there is a large shearing pattern of posterior fragments and the need for buttress plate fixation.

Other authors also have described the use of an anterolateral supra-fibular-head approach for managing a posterolateral tibial plateau fracture. Hu et al. performed the anterolateral supra-fibular-head approach and fixation with a lateral raft plate for the treatment of posterolateral fragments^14^. The results showed satisfactory knee joint function. It should be noted that the plates used in those studies may not have been specifically designed for anterolateral trans-fibular-head approach and may not have allowed more posterior placement of the plate. Meanwhile, plates were not anatomically matched and required pre-contouring, which may increase ligament tension and impede the functional recovery of the lateral collateral ligament complex postoperatively. In 2020, Hu designed a horizontal belt plate for the treatment of an isolated posterolateral fracture through the anterolateral supra-fibular-head approach, which was indicated for a pure articular depression fracture^18^. Giordano et al. also introduced the “hoop plating” technique, performed by placing a horizontal plate wrapped around the posterior rim of the tibial plateau^19^. However, when both the anterolateral and posterolateral columns of the tibial plateau are affected, the horizontal belt needs to be combined with other internal fixations, increasing surgical trauma and prolonging operation time. Several experts have proposed dual-plate fixation using a combined anterolateral and posteromedial inverted L-shaped approach, for plating anterolateral and posterolateral column fractures, with the patient in a floating position^20^. Among 41 cases, 3 patients experienced incision dehiscence or necrosis, and 2 patients suffered iatrogenic nerve injuries. Another combined approach reported by Zhang showed that 2 out of 17 cases experienced aseptic fat liquefaction after the operation^21^. Zhu et al. reported the use of a barrel hoop plate combined with a traditional lateral locking plate via a modified Frosch approach^22^. However, in this method, a conventional 2.7 radius T-plate needed to be cut and contoured to obtain the barrel hoop plate. A newly designed anatomical proximal lateral tibial rim plate, inserted via the anterolateral supra-fibular-head approach, demonstrates excellent biomechanical performance in our previous study^23^. In the herein study, the radiographic results of our patients indicated that the rear two screws can provide effective subchondral support for the articular surface fragments in Area I, and the other three screws can properly fix the fragments in Area II and Area III simultaneously. The plate body can buttress the fractured fragments, especially in cases of split wedge fragments of the lateral tibial plateau in the sagittal position. Our team suggests that it might be a good choice for an articular depression fracture combined with a minimal posterior cortex displacement that does not need reduction (Schatzker type II), a pure articular depression fracture (Schatzker type III), and Schatzker type VI involving the anterolateral and posterolateral columns. For a significant posterior cortex rupture and a posterolateral cortical wall requiring reconstruction, a direct posterolateral approach or Frosch approach is indicated. Besides, based on our previous research, it is noteworthy that when the distance from the apex of the fibular head to the lateral tibial plateau was < 8 mm in adults, the supra-fibular-head approach with our anatomical proximal lateral tibial rim plate was not indicated^24^.

The main limitations of the herein study were the relatively small number of patients and the lack of a control group. As a retrospective study, the inherent weaknesses and biases of such study designs are inevitable. In addition, a subdivision of patients based on posterolateral fracture morphology was missed here. Due to incomplete MRI examinations, possible intraarticular pathologies were not evaluated. Future prospective studies to assess more cases with a longer follow-up duration with a control group are needed.

## Conclusions

Our newly designed anatomical plate placed via the trans-supra-fibular approach successfully exposed and restored the fractured fragments and allowed the surgical procedure to be performed easily without requiring osteotomy. The plate comprises a long, narrow, transverse ring arm and can be used to support the posterolateral articular surface by providing two holes to affix the posterolateral fragments, resulting in satisfactory clinical outcomes.

## Data Availability

The datasets used and/or analyzed during the current study are available from the corresponding author or co-author on reasonable request.
